# Meetings of Societies

**Published:** 1941

**Authors:** 


### Meetings of Societies

### Bristol Medico-Chirurgical Society

By the courtesy of Surgeon Rear-Admiral Warren, O.B.E., a clinical meeting of the Bristol Medico-Chirurgical Society was held in the Recreation Hut at the Royal Naval Auxiliary Hospital, Barrow Gurney, on Thursday, 27th November, at 3.0 p.m. The President, Dr. Charles Corfield, was in the Chair.

Modern methods of treatment were illustrated by cases shown by Surgeon-Captains Critchley, Curran, and Lambert Rogers, and Surgeon-Commanders Oldham and Allison.

### British Medical Association

A meeting of the Bristol Division of the British Medical Association was held in the Reception Room at the University on Thursday, 20th November, at 3 p.m., when Dr. J. A. Nixon gave a British Medical Association lecture on "**The Treatment of Gas Casualties.**" Dr. H. M. Golding, D.F.C., was in the Chair.

